# Prognostic evaluation of preoperative systemic immune inflammatory index in patients with colorectal cancer

**DOI:** 10.3389/fonc.2023.1260796

**Published:** 2023-12-22

**Authors:** Tao Zhang, Yong chang Miao

**Affiliations:** Department of Gastrointestinal Surgery, Bengbu Medical College Lianyungang Clinical College, The Second People’s Hospital of Lianyungang, Lianyungang, Lianyungang, Jiangsu, China

**Keywords:** colorectal cancer, systemic immune-inflammation index, neutrophil-to-lymphocyte ratio, platelet-lymphocyte ratio, prognosis

## Abstract

**Objective:**

To investigate the impact of preoperative systemic immune inflammatory index (SII) on the clinical prognosis of patients undergoing colorectal cancer (CRC) surgery.

**Methods:**

One hundred and sixty CRC patients who underwent surgical treatment in our gastrointestinal surgery department from January 2019 to May 2023 were collected. ROC curves were applied to determine the sensitivity and specificity of SII, determine the optimal cut-off value into low SII and high SII groups, compare the clinicopathological data of SII patients in the two groups, and analyze the postoperative survival of patients in the two groups using Kaplan-Meier and Log-rank methods. Univariate and multifactor COX proportional risk regression models were used to analyze clinical prognostic factors.

**Results:**

The ROC curve showed that the area under the curve of SII for the evaluation of OS in CRC patients was 0.859, and the best cut-off value was 513.53. There was statistical significance (P < 0.05) in terms of tissue grading and diabetes mellitus in both groups. The Kaplan-Meier survival curves showed that the overall survival rates of the SII<513.53 group and the SII≥513.53 group were 50.88% (29/57) and 32.04% (33/103), and the overall survival rate of the SII<513.53 group was significantly higher than that of the SII≥513.53 group, and the difference was statistically significance (χ2 = 8.375, P=0.004). COX proportional risk regression showed that TNM stage, lymph node metastases, anastomotic fistula and SII were independent risk factors affecting postoperative survival in patients with CRC.

**Conclusion:**

Preoperative SII is an independent prognostic factor for CRC, which is simple, convenient, and non-invasive, and can be used to predict the prognosis of CRC patients.

## 1 Introduction

Colorectal cancer (CRC) is the third most common malignant tumor and the second leading cause of death in the world (1). In China, due to changes in living standards, lifestyles, and dietary habits in recent years, the incidence of CRC has been increasing, with colorectal cancer ranking third and fifth in terms of incidence and mortality rates, and in 2020, about 9.4 percent of deaths will be due to CRC disease (2). Currently, CRC is mainly surgical, but postoperative recurrence and metastasis severely limit the prognosis of CRC. In clinical practice, pathological type, tissue grading, and TNM stage are the most commonly used methods to predict the prognosis of CRC; however, prognostic heterogeneity still exists in patients with the same TNM stage (3). Inflammatory response is a key component of tumor development and a major cause of prognosis in tumor patients. There has been a marked increase in research on the relationship between inflammation and tumors, and combinations of these systemic inflammatory parameters, such as systemic immune-inflammatory index (SII), neutrophil-to-lymphocyte ratio (NLR), and platelet-lymphocyte ratio (PLR), are markers of active tumor inflammation (4, 5), which play an important role in promoting tumor progression. Generally, an increase in NLR, PLR, and SII is associated with a poor prognosis in tumor patients (6–11), and recent studies (12–14) have shown that the composite inflammatory markers of NLR, PLR, and SII can also be used as prognostic predictors for colorectal cancer patients. Although cancer is strongly associated with inflammation, the mechanisms linking patients with poor prognosis to elevated SII, NLR, and PLR need to be further investigated (15). In this paper, we retrospectively analyzed the clinical data of 160 cases of CRC patients admitted to our hospital from January 2019 to May 2023, and analyzed the effects of preoperative peripheral blood levels of SII, NLR, and PLR on the clinical prognosis of CRC patients, to provide a certain reference for CRC prognosis.

## 2 Information and methods

### 2.1 General information

**Figure 1:**
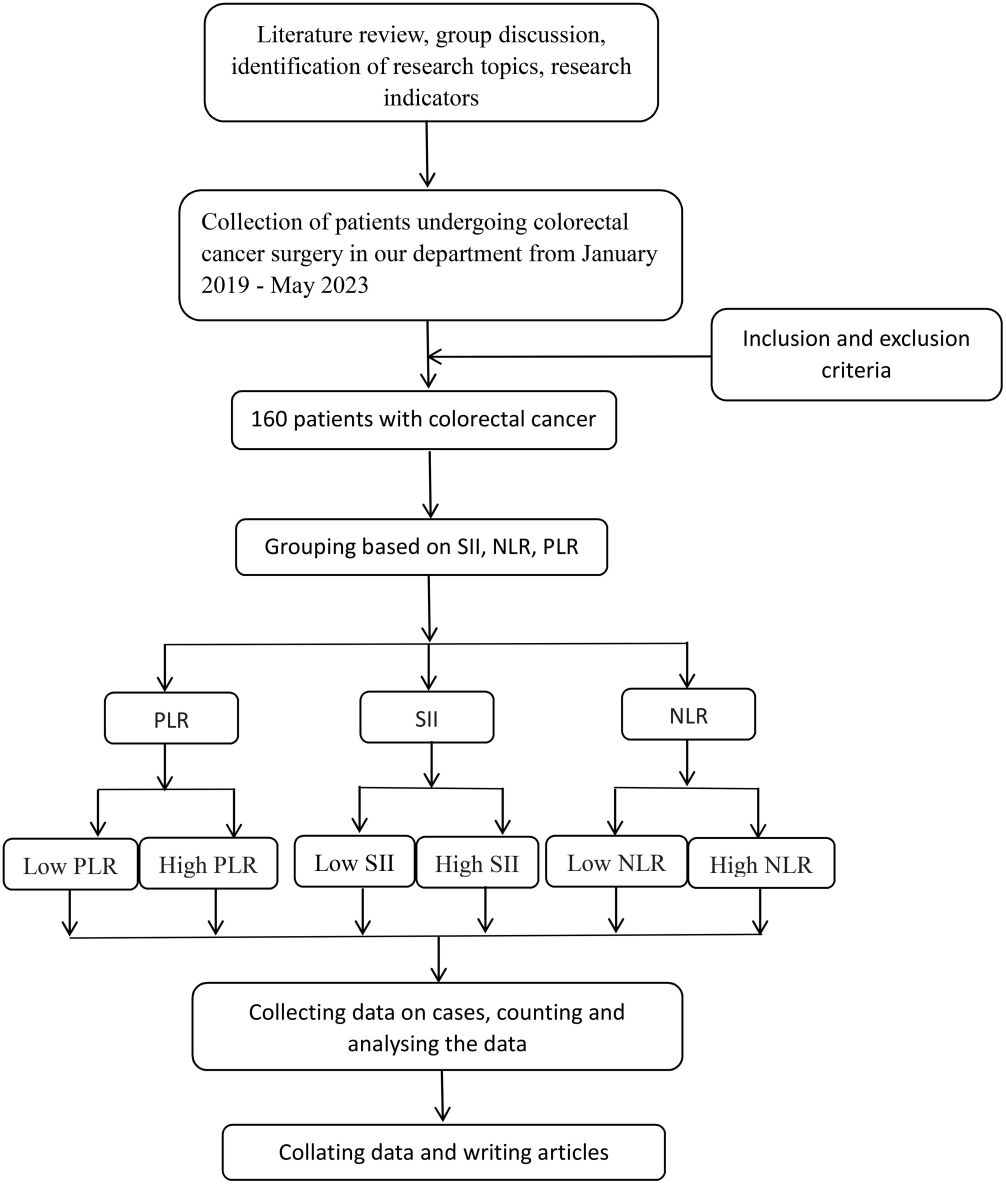
The flowchart of this paper is shown below. NLR, neutrophil-to-lymphocyte ratio; SII: systemic immune inflammatory index; PLR, platelet-to-lymphocyte ratio.

Retrospective analysis of clinicopathologic data of 160 patients who underwent CRC surgical treatment at the Department of Gastrointestinal Surgery of the Second People’s Hospital of Lianyungang City between January 2019 to May 2023. The flow chart is shown in **Figure 1**. Tumor TNM staging is based on the 8th edition of the American Joint Committee on Cancer (AJCC) TNM staging criteria for colorectal cancer.

Inclusion criteria: (1) All the selected patients underwent radical CRC surgery; (2) All the postoperative pathologies were CRC; (3) The patients did not undergo radiotherapy, chemotherapy and other immunotherapy before surgery.

Exclusion criteria: (1) Combination of other primary tumors; (2) Other serious infectious diseases, autoimmune diseases, etc. before surgery; (3) Blood diseases or history of trauma, blood transfusion, etc. before surgery; (4) Those who lost the visit after surgery; (5) Those who had missing pathological clinical data.

### 2.2 Methodology

General clinicopathological data such as age, gender, TNM stage, tumor diameter, diabetes mellitus, and coronary artery disease were collected, and patients were grouped according to the critical values of age and mean tumor diameter;


$$\begin{array}{l} \text{SII}=\text{neutrophil}(\times 10^{9}/\text{L})\times \text{platelet}(\times 10^{9}/\text{L})/\text{lymphocyte}(\times 10^{9}/\text{L});\\ \begin{array}{l} \text{NLR}=\text{neutrophil}(\times 10^{9}/\text{L})/\text{lymphocyte}(\times 10^{9}/\text{L});\\ \text{PLR}=\text{platelet}(\times 10^{9}/\text{L})/\text{lymphocyte}(\times 10^{9}/\text{L}). \end{array} \end{array}$$

### 2.3 Follow-up visits

A combination of outpatient, telephone, and We Chat was used for follow-up, with the first follow-up at 1 month after surgery, every 1 to 3 months during the first year after surgery, every 6 months during the second year after surgery, and annually from the third year after surgery onwards. The follow-up cut-off time was May 2023 or the patient’s death, and the overall survival (OS) was from the date of admission to the final follow-up cut-off time or the time of death.

### 2.4 Statistical methods

All data were analyzed using SPSS26.0 software for statistical analysis and processing of data, to establish the receive operating characteristic (ROC) curve, according to the Yo den index (Yo den index = sensitivity + specificity-1) the critical value corresponding to the maximum point was defined as the optimal truncation value of SII, NLR and PLR, and to calculate the sensitivity, sensitivity and area under the curve (AUC). The sensitivity, sensitivities and AUC were also calculated; Patients were grouped according to the SII optimal cutoff value; measurements were expressed using $\bar{\text{x}}\pm \text{s}$ , and counts were expressed as cases (%) using the χ2 test or Fishers exact test; Survival analysis was performed using the Kaplan-Meier method to plot survival curves and the Log-rank method to compare the survival differences between groups, to plot survival curves, and to compare the overall survival Overall survival (OS) of patients in different subgroups; All clinicopathological factors and SII were included in the univariate analysis, and multivariate analysis was performed for variables with meaningful differences, and the COX regression model was used to analyze the effect of each clinicopathological index on prognosis. Hazard ratio (HR) and 95% confidence interval (CI) were used to assess the relative risk, and P < 0.05 indicated a statistically significant difference.

## 3 Results

### 3.1 ROC curve analysis of SII, NLR, and PLR

**Figure 2:**
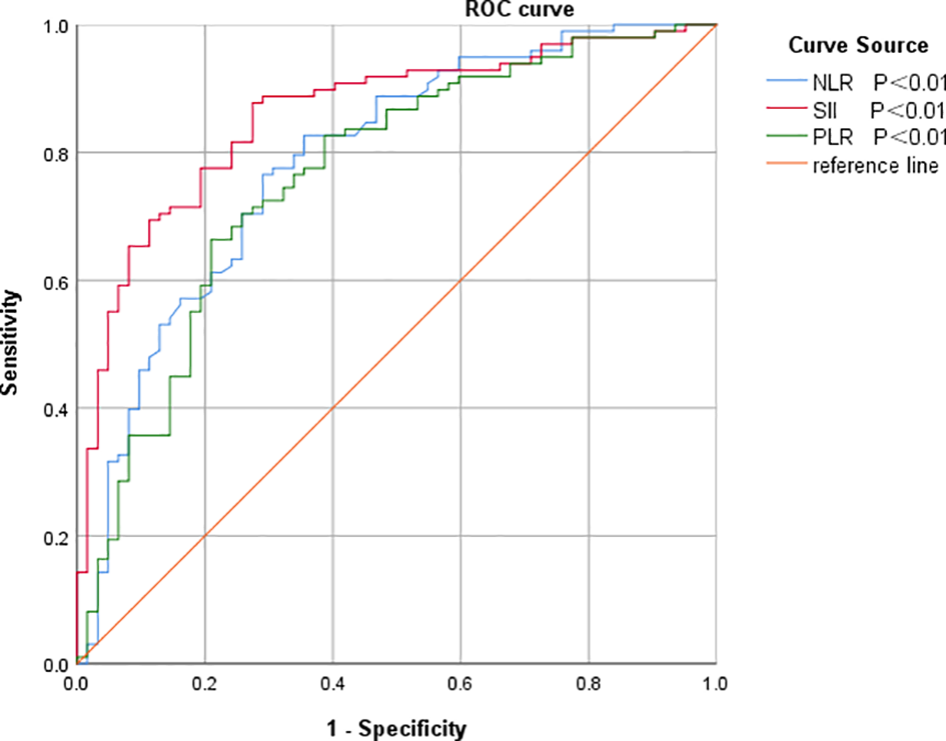
ROC curves of SII, NLR and PLR for predicting prognosis in colorectal cancer patients. NLR, neutrophil-to-lymphocyte ratio; SII, systemic immune inflammatory index; PLR, platelet-to-lymphocyte ratio.

The optimal intercept values for SII, NLR and PLR were selected based on the ROC curves, from which the patients were divided into two groups, high and low SII, high and low NLR, and high and low PLR, **Table 1** and **Figure 2**.

**Table 1:** Analysis of diagnostic efficacy of SII, NLR and PLR in predicting patients with colorectal cancer.

Research target	cut-off value	AUC (95% CI)	*P*	Sensitivity (%)	Specificity (%)
SII	513.53×10^9^/L	0.859 (0.800∼0.917)	<0.001	87.8	72.6
NLR	2.91×10^9^/L	0.788 (0.714∼0.862)	<0.001	76.5	71.0
PLR	141.42×10^9^/L	0.764 (0.687∼0.842)	<0.001	66.3	79.0

AUC, area under the curve; CI, confidence interval; NLR, neutrophil-to-lymphocyte ratio; SII, systemic immune inflammatory index; PLR, platelet-to-lymphocyte ratio.

### 3.2 Analysis of clinicopathological factors affecting SII, NLR and PLR

Clinicopathologic data of the whole group of 160 patients, the difference between SII and patients’ tissue grading and whether they had diabetes mellitus was statistically significant (P<0.05), The difference in terms of tissue grading between NLR and patients was statistically significant (P<0.05), The difference between PLR and patients in terms of gender and TNM staging was statistically significant (P<0.05) **Table 2**.

**Table 2:** Relationship between SII, NLR and PLR and clinicopathologic factors in patients with colorectal cancer.

Diagnostic trait	SII		*χ^2^*	*P*	NLR		*χ^2^*	*P*	PLR		*χ^2^*	*P*
	Low SII (n=103)	High SII (n=57)			Low NLR (n=67)	High NLR (n=93)			Low PLR (n=83)	High PLR (n=77)		
Sex, n (%)			0.249	0.618			0.284	0.594			0.158	0.691
Male	62 (60.2%)	32 (56.1%)			41(61.2%) (%)	53 (57.0%)			50 (60.2%)	44 (57.1%)		
Female	41 (39.8%)	25 (43.9%)			26 (38.8%)	40 (43.0%)			33 (39.8%)	33 (42.9%)		
Age, n (%)			0.799	0.371			0.070	0.791			0.194	0.660
<64years	53 (53.4%)	27 (47.4%)			31 (46.3%)	45 (67.2%)			37 (44.6%)	37 (48.1%)		
≥64years	50 (46.6%)	30 (52.6%)			36 (53.7%)	48 (32.8%)			46 (55.4%)	40 (51.9%)		
Tumor, n(%) (%) (years)diameter			0.030	0.864			1.402	0.236			0.340	0.560
<3.75cm	60 (58.3%)	34 (59.6%)			43 (64.2%)	51 (54.8%)			48 (57.8%)	41 (53.2%)		
≥3.75cm	43 (41.7%)	23 (40.4%)			24 (35.8%)	42 (45.2%)			35 (42.2%)	36 (46.8%)		
Tissue,n(%) classification hierarchy			0.595	0.441			1.941	0.164			0.083	0.774
Low level	66 (64.1%)	33 (57.9%)			42 (62.7%)	48 (51.6%)			45 (54.2%)	40 (51.9%)		
High level	37 (35.9%)	24 (42.1%)			25 (37.3%)	45 (48.4%)			38 (45.8%)	37 (48.1%)		
TNM staging, n (%)			2.003	0.572			0.825	0.844			0.482	0.923
I	19 (18.4%)	10 (17.5%)			12 (17.9%)	17 (18.3%)			16 (19.3%)	12 (15.6%)		
II	41 (39.8%)	20 (35.1%)			26 (38.8%)	30 (32.3%)			28 (33.7%)	27 (35.1%)		
III	30 (29.1%)	15 (26.3%)			19 (28.4%)	31 (33.3%)			27 (32.5%)	25 (32.5%)		
III	13 (12.6%)	12 (21.1%)			10 (14.9%)	15 (16.1%)			12 (14.5%)	13 (16.9%)		
Diabetes, n (%)			2.036	0.154			0.312	0.577			0.044	0.834
With	53 (51.5%)	36 (63.2%)			39 (58.2%)	50 (53.8%)			52 (62.7%)	47 (61.0%)		
Without	50 (48.5%)	21 (36.8%)			28 (41.8%)	43 (56.2%)			31 (37.3%)	30 (39.0%)		
Coronary heart, n (%)			1.039	0.308			0.597	0.440			0.305	0.581
With	42 (40.8%)	28 (49.1%)			24 (35.8%)	43 (46.2%)			37 (44.6%)	31 (40.3%)		
Without	61 (59.2%)	29 (50.9%)			43 (64.2%)	60 (53.8%)			46 (55.4%)	46 (59.7%)		
Lymph node metastases, n (%)			0.682	0.409			0.391	0.532			0.020	0.887
With	60 (58.3%)	37 (64.9%)			45 (67.2%)	58 (62.4%)			53 (63.9%)	50 (64.9%)		
Without	43 (41.7%)	20 (35.1%)			22 (32.8%)	35 (37.6%)			30 (26.1%)	27 (35.1%)		
Intestinal obstruction, n (%)			0.379	0.538			0.010	0.922			0.004	0.951
With	33 (32.0%)	21 (36.8%)			25 (37.3%)	34 (36.6%)			23 (27.7%)	21 (37.7%)		
Without	70 (68.0%)	36 (63.2%)			42 (62.7%)	59 (63.4%)			60 (62.3%)	56 (72.3%)		
Nerve invasion status, n (%)			2.760	0.097			0.009	0.926			0.462	0.496
With	22 (21.4%)	19 (33.3%)			17 (25.4%)	23 (24.7%)			27 (32.5%)	29 (37.7%)		
Without	81 (78.6%)	38 (66.7%)			50 (74.6%)	70 (75.3%)			56 (67.5%)	48 (62.3%)		
Vascular thrombus,n (%)			2.123	0.145			0.043	0.836			1.384	0.239
With	25 (24.3%)	20 (35.1%)			22 (32.8%)	32 (46.2%)			25 (30.1%)	30 (39.0%)		
Without	78 (75.7%)	37 (64.9%)			45 (67.2%)	61 (53.8%)			58 (69.9%)	47 (61.0%)		
Anastomotic fistula,n (%)			1.358	0.244			0.105	0.746			0.498	0.480
With	19 (18.4%)	15 (26.3%)			20 (29.9%)	30 (32.3%)			20 (24.1%)	15 (19.5%)		
Without	84 (81.6%)	42 (73.7%)			47 (70.1%)	63 (67.7%)			63 (75.9%)	62 (80.5%)		

NLR, neutrophil-to-lymphocyte ratio; SII, systemic immune inflammatory index; PLR, platelet-to-lymphocyte ratio; TNM, tumor-node-metastasis.

### 3.3 Analysis of survival outcomes

**Figure 3:**
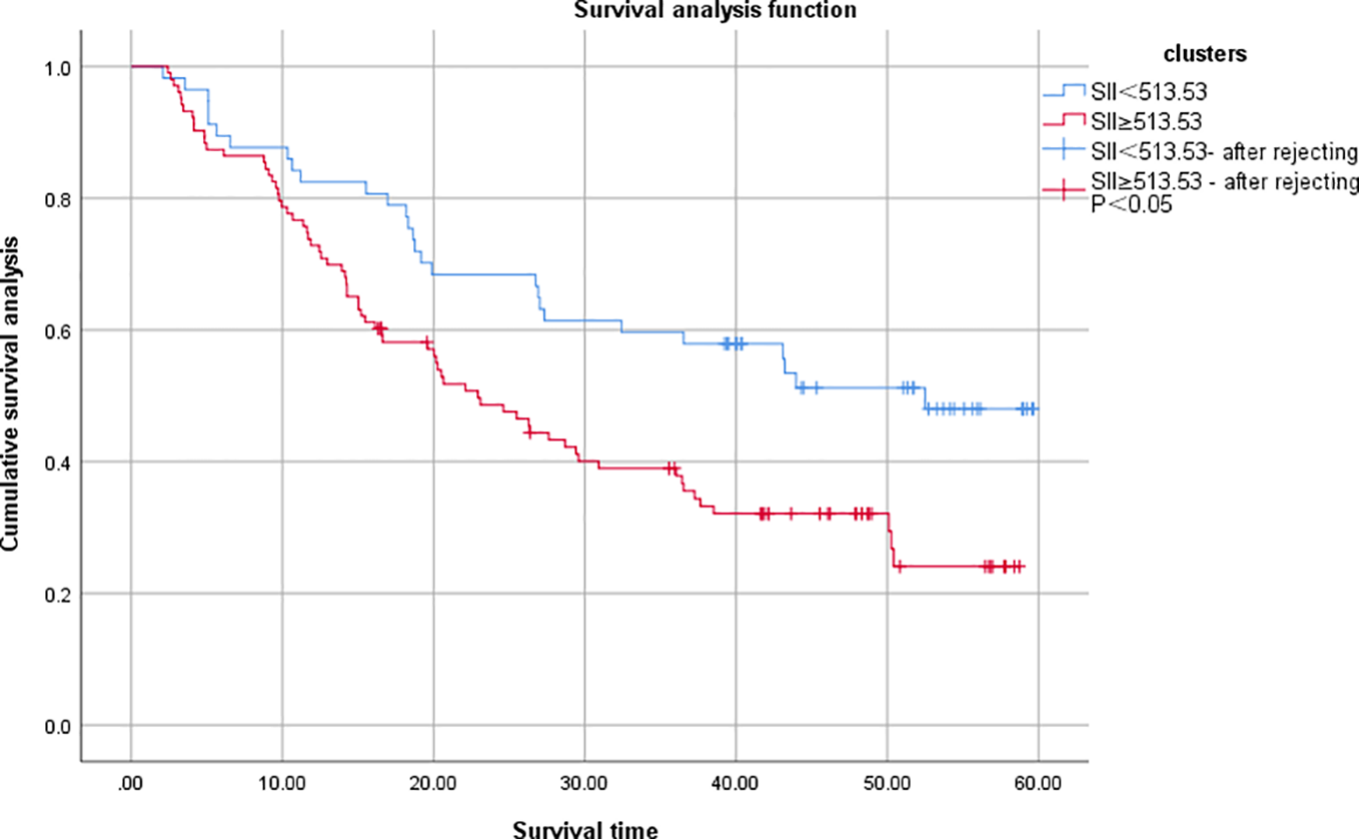
Comparison of OS between two groups of colorectal cancer patients. SII, systemic immune inflammatory index.

Survival analysis was complete in 160 patients with a mean follow-up of 29.25 (2-60) months, with 62 (38.75%) survivors and 98 (61.25%) deaths by the time of the last follow-up. The overall survival rates of the SII <513.53 and SII≥513.53 groups were 50.88% (29/57) and 32.04% (33/103), and the overall survival rate of the SII<513.53 group was significantly higher than that of the SII≥513.53 group, and the difference was statistically significant (χ2 = 8.375, P=0.004), **Figure 3**. The results of univariate analysis showed statistically significant differences in terms of tumor diameter, TNM stage, lymph node metastases, anastomotic fistula, intestinal obstruction, vascular thrombus, nerve invasion status and SII; the inclusion of univariate significant factors in the COX multifactorial analysis showed that patient’s TNM stage, lymph node metastases, anastomotic fistula and SII were independent risk factors affecting the prognosis of CRC patients **Table 3**.

**Table 3:** Unifactorial and multifactorial analyses affecting the prognosis of patients with colorectal cancer.

Factor	Single factor analysis			Multiple-factor analysis		
	*HR*	(95% *CI*)	*P*	*HR*	(95% *CI*)	*P*
Sex (0/1)	1.197	0.788-0.819	0.399			
Age/years (0/1)	0.915	0.616-1.553	0.410			
TD/cm (<3.75cm/≥3.75cm)	1.847	1.235-2.762	0.003	1.650	0.675-1.720	0.086
TC (0/1)	0.809	0.554-1.310	0.480			
TNM staging (I/II/III/IV)	0.592	0.513-1.065	0.021	0.171	0.202-0.490	0.001
Diabetes (with/without)	0.712	0.500-1.108	0.071			
Coronary heart (with/without)	1.513	0.812-2.301	0.132			
Lymph node metastases (with/without)	1.522	1.432-1.585	<0.001	1.234	1.146-1.217	0.001
Intestinal obstruction (with/without)	1.603	1.123-2.292	0.009	1.813	1.186-2.641	0.106
Nerve invasion status (with/without)	0.734	0.062-0.884	0.001	0.875	0.843-1.186	0.205
Vascular thrombus(with/without)	1.699	1.572-1.737	<0.001	1.252	1.145-1.372	0.301
Anastomotic fistula(with/without)	2.760	2.203~3.173	<0.001	1.255	1.246-1.714	0.001
NLR (<2.91/≥2.91)	1.077	1.028-1.130	0.002	1.347	0.673-1.904	0.260
PLR (<141.42/≥141.42)	1.044	0.743-1.384	0.014	1.311	0.062-2.677	0.201
SII (<513.53/≥513.53)	2.799	1.790-4.376	<0.001	2.444	1.421-3.003	0.001

HR, hazard ratio; CI, confidence interval; TNM, tumor-node-metastasis; NLR, neutrophil-to-lymphocyte ratio; SII, systemic immune inflammatory index; PLR, platelet-to-lymphocyte ratio; TD, tumor diameter; TC, tissue classification.

Variable assignment: sex (male=0, female=1); age (<64 years=0, ≥64 years=1); Tissue classification (low level=0, high level=1).

## 4 Discussion

CRC is one of the most common malignant tumors of the gastrointestinal tract, with inconspicuous early manifestations, and most of them are already in the middle and late stages when they are detected. In recent years, the incidence of CRC has been increasing year by year, and it is mostly found in middle-aged and elderly people. Due to the highly invasive and malignant nature of the tumor, it leads to poor prognosis, high mortality and short survival time. Currently, surgery is the main treatment for CRC patients, and postoperative adjuvant radiotherapy, targeted therapy and immunotherapy and other comprehensive treatment methods have made great progress in treatment, but the survival time after surgery is still not significantly improved. Therefore, it is very meaningful to actively seek and explore the influencing factors that predict the prognosis of CRC patients.

Inflammation is an important component of the tumor microenvironment, and peripheral blood inflammatory cells can directly or indirectly interact with tumor cells, thereby promoting tumor cell proliferation, migration and invasion, and inhibiting apoptosis (16, 17). In recent years, the role of inflammatory factors in malignant tumors has received increasing attention, including neutrophils, platelets and lymphocytes, which are major factors in angiogenesis, invasion and metastasis in the tumor-associated inflammatory microenvironment (18–20). Neutrophils can directly promote tumor proliferation, metastasis, and local angiogenesis through the secretion of a variety of pro-angiogenic factors, and they also play an important role in the migration and invasion of circulating tumor cells (21). Yang et al. (22) showed that a high serum neutrophil count was associated with OS and progression-free survival in patients with metastatic colorectal cancer Ras wild type. There is growing experimental and clinical evidence that platelet activation can act as a chemotactic agent for cancer cells, inducing optimal conditions for the formation of metastatic foci, and that platelets promote the survival of cells with high metastatic potential during their haemotransport (23). Lymphopenia is usually accompanied by leukocytosis and thrombocytosis, which may help tumor cells evade immune surveillance and prevent damage caused by an autoimmune response of cytotoxic T cells (20). SII first studied by Hu et al. (24) in hepatocellular carcinoma, is a new inflammatory index based on neutrophils, platelets and lymphocytes, which combines the three types of immune-inflammatory cells mentioned above, and can comprehensively reflect the balance between immune and inflammatory responses in patients with tumors. Elevated levels of SII are mostly caused by the elevation of neutrophils and platelets, and the reduction of lymphocyte levels, suggesting that patients have increased inflammatory responses and weakened immune responses, indicating a higher risk of tumor recurrence and a worse prognosis. Elevated SII is mostly caused by elevated neutrophils and platelets and decreased lymphocyte levels, suggesting that the patient’s inflammatory response is enhanced and the immune response is weakened, indicating that the patient has a higher risk of tumor recurrence and a worse prognosis.

The objective of this study was to discuss the clinicopathological and prognostic value of systemic inflammatory markers, including SII, NLR, and PLR, in patients with colorectal cancer, and to compare their predictive accuracy. After analyzing the predictive value of inflammatory indicators for CRC by ROC curve, the best cut-off value was determined, and it was found that the best cut-off values of SII, NLR and PLR for CRC prediction were 513.53, 2.91, and 141.42, with the AUCs of 0.859, 0.788, and 0.764, respectively, which indicated that the three inflammatory indicators had good predictive value for CRC, but the predictive value of SII was higher than that of NLR and PLR. value was higher than NLR and PLR.According to the best cut-off value of SII, all patients were divided into a low SII group and a high SII group.Kaplan-Meier survival analysis found that the OS of the low SII group was significantly better than that of the high SII group, and the survival period was longer, which was consistent with the studies of Chen et al. (25) and Xie et al. (26). The results of univariate and multifactorial analyses showed that patients’ TNM stage, lymph node metastases, anastomotic fistula and SII were independent risk factors affecting the prognosis of CRC patients.

In the present study, the cut-off values of SII, NLR and PLR for predicting CRC were 513.53, 2.91 and 141.42, respectively, which were different from the cut-off values of other studies. There are a number of reasons for the difference regarding the cut-off values. Firstly, the present study was retrospective, single center, small sample size and there may be confounding errors; Secondly, some studies have taken different times for blood routines, resulting in potentially large differences in some indicators of inflammation; Again, some studies have inconsistent inclusion and exclusion criteria, which may also affect the cut off values; Finally, in patients with colorectal cancer, differences in surgical approach and operator experience can also lead to some differences in cut off values. Taken together, these can cause some differences in cut-off values.

The author’s analysis of the predictive value of SII for tumor recurrence may be based on the following aspects. (1) Neutrophils may infiltrate into the tumor microenvironment and become tumor-associated neutrophils, releasing chemical and cytokines associated with tumor proliferation and metastasis, such as vascular endothelial growth factor (VEGF), elastase and matrix metalloproteinases (27). (2) Lymphocytes, as the most important immune cells of the body, when stimulated by antigens, T-lymphocytes will trigger specific immune responses and participate in the immune response to tumors, thus inhibiting tumor growth and improving the prognosis of cancer patients (28). Therefore, the lower the lymphocyte level, the worse the patient’s immune ability; while elevated neutrophils will inhibit the activation of T-lymphocytes, leading to a reduction in the body’s anti-tumor ability. (3) Platelets originate from megakaryocytes in the bone marrow, which not only have the function of coagulation, but are also related to the occurrence and development of tumors. Tumor cells can damage vascular endothelium, activate platelets to release vascular endothelial growth factor to repair endothelial cells, promote the formation of tumor neovascularization and the adhesion of tumor cells to the vascular wall, resulting in tumor cell proliferation and metastasis, and thus forming a vicious cycle (29).Therefore, higher neutrophil and platelet counts and lower lymphocyte counts, i.e. high SII tend to suggest that patients are at higher risk of tumor recurrence and have a worse prognosis.

## 5 Conclusion

In conclusion, preoperative SII can be used as an effective predictor for determining the prognosis of CRC patients, which is worth promoting and applying in clinical practice. However, this study still has some shortcomings, firstly it is a single-center, small-sample retrospective study and there may be a case selection bias; Secondly, this study did not analyze whether the occurrence of postoperative complications had an impact on survival; Finally, different surgical experiences of surgeons can also lead to differences in postoperative tumor recurrence. Therefore, prospective, large sample and multi-center studies are still needed to verify the value of SII in predicting tumor recurrence in CRC patients.

## Data availability statement

The original contributions presented in the study are included in the article/supplementary material. Further inquiries can be directed to the corresponding author.

## Ethics statement

The studies involving human participants were reviewed and approved by Medical Ethics Committee of the Second People’s Hospital of Lianyungang. The studies were conducted in accordance with the local legislation and institutional requirements. Written informed consent for participation was not required from the participants or the participants’ legal guardians/next of kin in accordance with the national legislation and institutional requirements. Written informed consent was obtained from the individual(s) for the publication of any potentially identifiable images or data included in this article.

## Author contributions

TZ: Conceptualization, Writing – original draft, Writing – review & editing. YM: Supervision, Writing – review & editing, Funding acquisition.
